# Alkali Polymer Flooding of a Romanian Field Containing Viscous Reactive Oil

**DOI:** 10.3390/polym16060854

**Published:** 2024-03-20

**Authors:** Eugen Hoffmann, Rafael E. Hincapie, Ante Borovina, Torsten Clemens, Muhammad Tahir, Markus Lueftenegger, Jonas Wegner

**Affiliations:** 1 HOT Microfluidics GmbH, 38640 Goslar, Germany; jwegner@hoteng.com; 2 OMV Exploration and Production GmbH, 1020 Vienna, Austria; rafaeleduardo.hincapiereina@omv.com (R.E.H.); ante.borovina@omv.com (A.B.); torsten.clemens@omv.com (T.C.); muhammad.tahir@omv.com (M.T.); 3 OMV Petrom S.A., 013704 Bucharest, Romania; markus.lueftenegger@petrom.com

**Keywords:** alkali-polymer, IFT, Micromodel, coreflood, saponification, emulsions

## Abstract

The study demonstrates the significant enhancement in oil production from a Romanian oil field using alkali–polymer (AP) flooding for reactive viscous oil. We conducted comprehensive interfacial tension (IFT) measurements across various alkali and AP concentrations, along with phase behavior assessments. Micromodel flooding experiments were used to examine pore-scale effects and select appropriate chemical concentrations. We tested displacement efficiency at the core level and experimented with different sequences and concentrations of alkali and polymers to minimize costs while maximizing the additional recovery of reactive viscous oil. The IFT analysis revealed that saponification at the oil–alkali interface significantly lowers IFT, but IFT gradually increases as soap diffuses away from the interface. Micromodels indicated that polymer or alkali injection alone achieve only minimal incremental recovery beyond waterflooding. However, AP flooding significantly enhanced incremental oil recovery by efficiently moving the mobilized oil with the viscous fluid and increasing exposure of more oil to the alkali solution. Coreflood experiments corroborated these findings. We also explored how divalent cations influence polymer concentration selection, finding that softening the injection brine significantly increased the viscosity of the AP slug.

## 1. Introduction

The extraction of heavy, viscous oil is hindered by low rates of recovery due to unfavorable mobility ratios. By increasing water injection rates and decreasing the distance between wells, the efficiency of oil recovery through waterflooding is enhanced for such deposits [1]. Additionally, employing chemical enhanced oil recovery (EOR) techniques can lead to significant improvements in recovery rates beyond those achievable with waterflooding alone. The effectiveness of polymer injection in enhancing the recovery of heavy oil has been confirmed through various laboratory studies (e.g., Levitt et al. [2]), displacement efficiency calculations [3], and micromodel investigations (e.g., Buchgraber et al. [4]), culminating in economically successful field applications of polymer flooding in heavy oil fields [5,6,7].

For further improvement of recovery efficiencies obtained from polymer flooding, the enhancement of microscopic displacement efficiency is crucial. This can be realized through the generation of Winsor III type emulsions, characterized by ultra-low interfacial tensions, thereby facilitating the mobilization of residual oil within porous media (e.g., Fortenberry et al. [8], Torrealba and Johns [9], and Abdelfatah et al. [10]). For oils with a high total acid number (TAN) and low to medium viscosity ranges, alkali injection serves as a method to catalyze in situ soap formation at the interfaces of residual oil post-waterflooding, as reported by deZabala and Radke [11], Sharma et al. [12], and Magzymov et al. [13]. The generated soaps can effectively mobilize residual oils, particularly when polymers are incorporated to boost the local capillary number, enhancing mobilization (e.g., Schumi et al. [14] and Hincapie et al. [15]).

Alkali polymer (AP), as demonstrated in micromodels and corefloods, proves to be a cost-effective method for augmenting oil recovery from reactive medium viscous oil by enhancing the local capillary number and mobilizing oil (e.g., Schumi et al. [14] and Hincapie et al. [15]). The positive effects of alkali on polymer solution viscosity development, adsorption, and injectivity, and the long-term stability of polymers in alkali solutions were explored by Nurmi et al. [16], indicating additional economic benefits of AP flooding over polymer flooding. While various chemical enhanced oil recovery (EOR) projects have been successfully field-tested and implemented for light oils (e.g., Guo et al. [17], Pandey et al. [18], and Volokitin et al. [19]), heavy oils require additional chemicals to polymers for increased production over waterflooding. Alkali and surfactant injection induces emulsion formation and channel blockage in waterflooded cores and micromodels (e.g., Bryan et al. [20], Dong et al. [21], Xie et al. [22], and Xiao et al. [23]). The implementation of alkali surfactant polymer (ASP) flooding in heavy oil fields has shown substantial incremental oil recovery, indicating successful translation of promising laboratory tests to field conditions (e.g., McInnis et al. [24], Watson et al. [25], and Al Marouqi et al. [26]), although field cases suggest that operational conditions are more challenging for ASP projects than for polymer floods (Delamaide et al. [27]).

This paper explores the physio-chemical processes and recovery efficiency of alkali flooding and the alkali polymer flooding of heavy oil under unstable flooding conditions using corefloods and micromodels. It investigates effects at oil–water interfaces, displacement efficiencies, and the role of polymers in these processes. The paper provides an overview of the approach, discusses materials and methods, and describes micromodel experiments and out-crop and sandpack flooding experiments using reservoir material. The novelty lies in the examination of the reservoir studied and the observation of new mechanisms of alkali and polymer at the pore scale using a micromodel.

## 2. Experimental Approach & Flood Media

Numerous experiments and analyses were conducted to examine the effects of alkali/alkali–polymer injection in a system with high TAN dead oil, with various steps:

1. Evaluate fluid–fluid interactions by performing interfacial tension (IFT) and phase behavior measurements.
2. Carry out micromodel flooding to further understand displacement efficiency effects (mechanisms at pore scale). A naturally hydrophilic micromodel was designed with dimensions of 120 × 45 mm resembling Bentheimer sandstone characteristics.
3. Conduct coreflooding experiments to better understand the displacement efficiency at a core scale level.

Reservoir Data: We have evaluated the feasibility of alkali–polymer in a heavy oil field in eastern Romania. Reservoir ML contains ~50 active wells and reservoir thickness is ~10 m in a shallow marine environment. The section consists of three sands, from which sands 1 and 2 are often interlinked. The current reservoir pressure is ~36 bar and oil viscosity 100 mPa·s. ML is characterized by sequences of clastic sediments with an initial oil–water-contact located at 525 m TVD Sub Sea. The average permeability and porosity are around 1 Darcy and 30%, respectively, with a temperature of ~35 °C. In particular, the reservoir heterogeneity comes from the variation of two areas, the main reservoir with k values between 0.8 and 1 D and the shaly reservoir between 0.2 D and 0.4 D.

**Figure 1:**
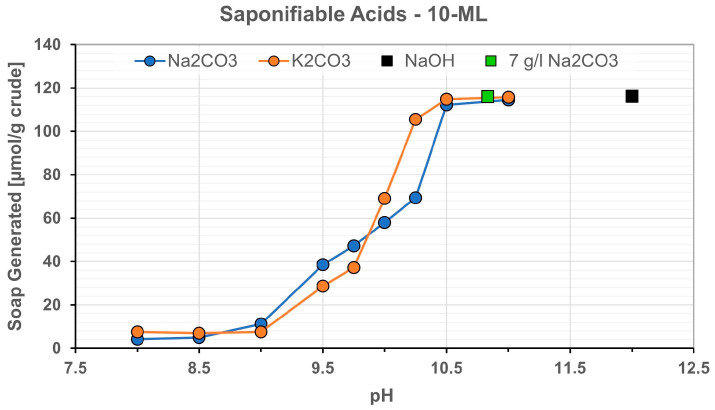
Soap generated (titration) at given pH for the 10-ML crude oil used in this work. For the titration, the oil sample was saponified (contacted) entirely with alkali, and the excess is measured by titration (in mg KOH/g).

Oil Data: For this study, we used oil from the well 10-ML. The oil from the ML reservoir is biodegraded. This could be beneficial since biodegradation often leads to the formation of oils that are reactive with alkali. The data are summarized in Table 1. The TAN number of the oil amounts to 5.67 mg KOH/g and the saponifiable acids around 116 µmol/g, as determined by the method described by Southwick et al. [28], shown in Figure 1 and measured by soap generated by titration at a given pH for the 10-ML crude oil. For experimental application, a mixture was used in order to match the reservoir viscosity, 100 mPa·s at 35 °C, using 10-ML oil + 4% cyclohexane. Note that the oil samples as taken from the field contained 45% water and it was only possible to get it down to 40%. Therefore, the original oil samples visibly contained emulsions.

Brines: Details on the brine composition used in this study are listed in Table 2. The reservoir brine composition, here named IWS-1010, was used as a synthetic brine for core initialization, injection, and partial fluid preparation, containing 15.84 g/L TDS. As reported by Hincapie et al. [15], to mimic the salt content and the pH versus alkali concentration behavior of the actual injection brine IWS-1010, the brine in which the chemicals are prepared for injection (SBB-1010) included 15.84 g/L TDS, with 0.46 g/L NaHCO_3_ used as buffer capacity to avoid solid precipitation. Brine viscosity at 35 °C was measured to be 0.5 mPa·s.

Alkali and Polymer: As pointed out by Schumi et al. [14] and Hincapie et al. [15], for the purpose of cost reduction and the prevention of the risk of silica scale formation in the production wells, Na_2_CO_3_ was used as the alkali. Various concentrations (5 g/L, 7 g/L, and 15 g/L) of Na_2_CO_3_ were deployed to identify the best cost/performance ratio. As an orientation, the solution containing 7.5 g/L Na_2_CO_3_ yielded a pH value of 10. Hydrolyzed polyacrylamide (HPAM) FP 6030 S with a 20–30% hydrolysis degree was used as polymer, being a high molecular weight polyacrylamide (HPAM) with 25–30 Megadalton molecular weight. FP 6030 S is a homopolymer post hydrolyzed of Acrylamide for T < 75 °C and low hardness. Two polymer concentrations were used during the experiments, namely 1.5 g/L and 2.2 g/L, leading to various viscosities. Viscosity data will be shown for each specific experiment in a later section.

Outcrop Cores: Bentheimer sandstone cores (composition listed in Table 3) were used for flooding experiments. A diameter of 2.96 cm, a length of 30.03 cm, porosity of about 0.24, and brine permeability with an average around 2250 mD were measured. Possible heterogeneities were ruled out using the “Phoenix Nanotom M (Waygate Technologies, Wunstorf, Germany)” nano CT scanner. Potassium Bromide (KBr) conservative tracer experiments depicted a dispersivity of about 0.05 cm. The conservative tracer did not cause any changes to the phase behavior and was injected at a concentration of 1 g/L. 

Reservoir Material: Sandpack experiments were conducted using reservoir rock material, which was homogenized into a uniform sand pile. Material from two primary reservoir sections, referred to as the main reservoir and shaly reservoir, was utilized (refer to Table 3). Details regarding sandpack characteristics such as porosity and permeability for each specific case are provided in the subsequent section.

## 3. Methods

Interfacial Tension (IFT) and Phase Behavior Assessments: Our methodology for conducting IFT and phase behavior experiments was adapted from the procedures outlined by Hincapie et al. [15] and Saleh et al. [29]. IFT measurements were carried out using a KRUSS spinning drop tensiometer operating at 35 °C and 4000 rpm, following the principles described by Sharma et al. [12]. Phase behavior tests, including the volumes of oleic and aqueous phases, were monitored over a two-month period using a 50/50 ratio (either brine, polymer brine, or alkali–polymer brine as aqueous solution). Here, “oleic phase” refers to the dead oil sample without cyclohexane. These experiments were conducted at 35 °C and atmospheric pressure using dead oil samples.

**Figure 2:**
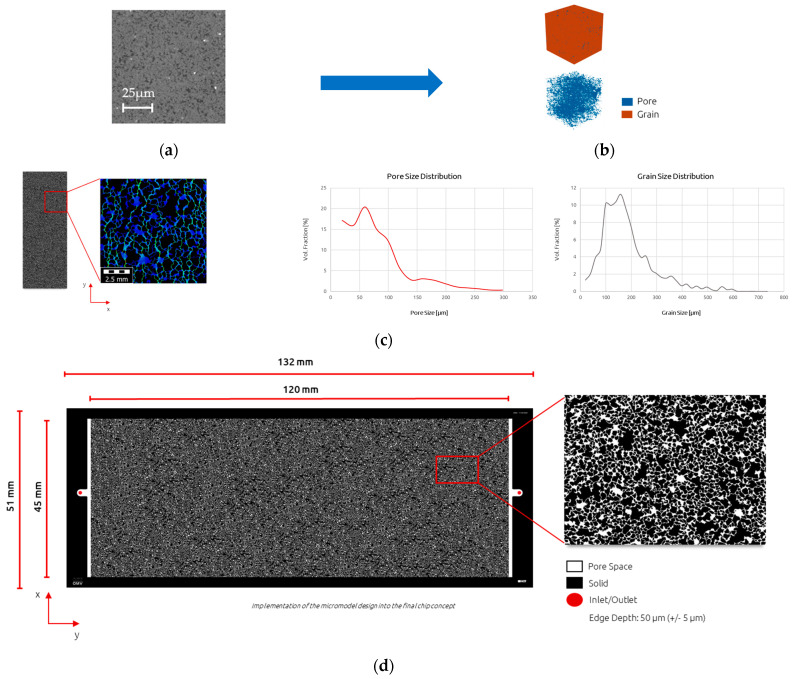
Step by step micromodel generation workflow utilized in this work. (**a**) Generation of micro−CT scans. (**b**) Conversion of grayscale image stack to 3D model. (**c**) Upscaling to desired dimensions, pore-scale simulation and determination of properties (φ, k, pore and grain size distribution, etc.). Permeability simulations yielded a value of 1.6 D. (**d**) The final design of 120 × 45 mm micromodel.

Micromodel Generation and Configuration: The utilization of micromodel flooding experiments provides an efficient and visually impactful method for initially screening EOR solutions. In this study, we designed and fabricated a micromodel with dimensions of 120 × 45 mm (refer to Figure 2). While the experimental setup remained the same, the design presented here differs from the microchips typically employed in previous studies by Schumi et al. [14], Borovina et al. [30], and Hincapie et al. [15]. Notably, our setup incorporates an automated scheduling feature, enabling the programming of experiment sequences to mitigate human error and ensure hardware reproducibility. An advantageous aspect of this new enlarged micromodel design is the increased distance between the injection and contact areas, allowing for the investigation of unstable displacement phenomena on a larger scale compared to smaller micromodels.

**Figure 3:**
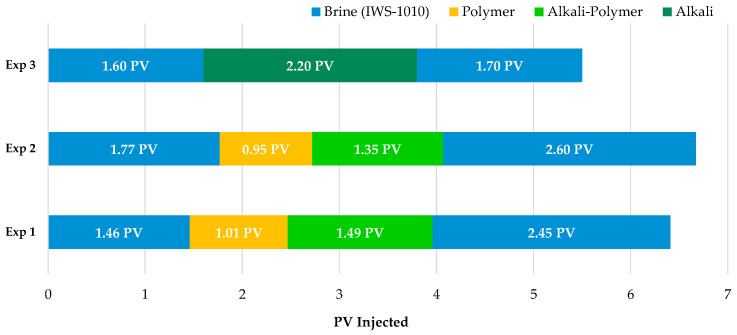
Overview of the injection sequences in micromodel experiments. In the instance of brine injection, IWS-1010 served as both the initial and final slug.

Micromodel Flooding Experiments—Sequence of Events: The micromodel experiments followed a four-step injection procedure: brine, chemical slug 1, chemical slug 2, and brine. A consistent interstitial flow velocity of 1 ft/day was maintained throughout all steps. Initially, synthetic brine was injected up to 1.5 pore volumes. Subsequently, polymer solution injection was carried out up to 1 pore volume, followed by alkali–polymer solution injection up to 1.5 pore volumes. Throughout each experiment, elapsed time, differential pressure, and temperature values at four positions (top, bottom, left, and right position of the respective micromodel to avoid temperature gradient) were monitored, alongside the capture of high-resolution images. Table 4 summarizes the parameters utilized during the micromodel floods, while Figure 3 illustrates the injection sequences employed. In the case of brine injection, IWS-1010 was utilized for both the initial and final slug.

Core Flooding Experiments: Seven corefloods were conducted using analogue sandstone rock material with properties resembling those of the targeted reservoir area. Additionally, two types of unconsolidated reservoir rock material (from the main reservoir and shaly reservoir) were utilized as sandpacks to compare and validate the results obtained from the analogue rock experiments. All coreflood experiments were conducted using the same setup as reported by Schumi et al. [14] and Hincapie et al. [15]. 

For core/sandpack initialization, adapted routine core analysis techniques were followed to assess the properties of the analogue rock material. The dry and cleaned core was placed into a holder and subjected to a radial confining pressure of 30 bar(g) with a pore pressure of 10 bar(g). Continuous injection of CO_2_ into the dry and cleaned core was performed for a minimum of ten minutes to remove oxygen. Subsequently, the core was flow-through saturated with synthetic formation brine (IWS-1010) at a pore pressure of at least 5 bar(g) while the temperature of the oven surrounding the core holder was gradually increased to 35 °C. At stabilized temperature, permeability to brine was measured at three appropriate flow rates. Following this, pressure and temperature were reduced to ambient conditions, and the sample was unloaded from the core holder and weighed.

The pore volume of the core sample was calculated using the Archimedes method. Subsequently, the sample was initialized with dead crude oil until a target water saturation of approximately 20% was achieved, representing the initial water saturation. Produced fluids were collected, and the initial water saturation was confirmed using Dean–Stark. Effective permeability to oil was determined at three suitable injection rates, ensuring that the differential pressure remained below the desaturation pressure. Careful monitoring of the pressure differential was conducted to identify any potential plugging effects induced by the injected oil.

**Figure 4:**
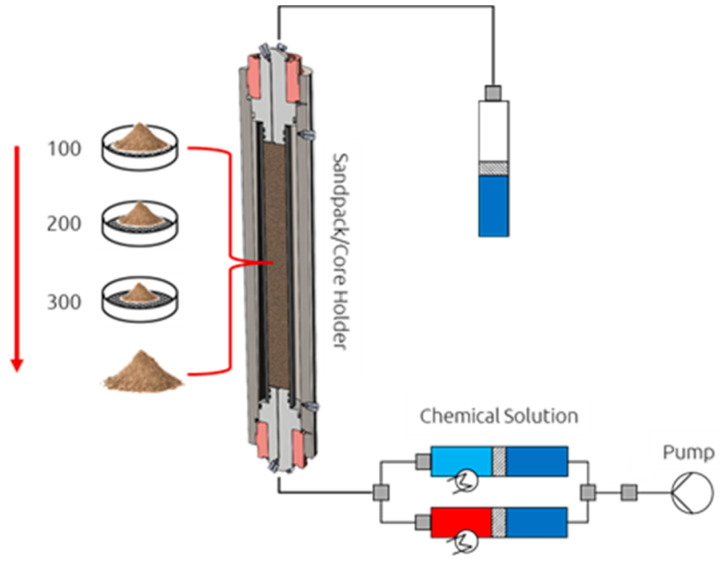
Schematic illustration of sand material used for preparation of a sandpack.

Sandpack Preparation: A procedure for preparing sandpacks was adapted for experiments involving unconsolidated rock material. Reservoir rock was crushed into a consistent sand pile, which was then placed into a Soxhlet apparatus. The rock material underwent cleaning for a minimum of four days using an 80/20 mixture ratio of chloroform and methanol to extract any remaining salt and hydrocarbons. Subsequently, the cleaned sand was dried at temperatures ranging from 50 to 60 °C for several days and passed through sieves with various mesh sizes (100, 200, and 300 µm) until uniformly distributed sand clusters were attained. These clusters served as the foundation for the sandpack mixtures, ensuring matching of permeability of the targeted reservoir. A schematic illustration of the sandpack preparation process in the core holder cell is depicted in Figure 4.

Every sandpack contained 350 g of reservoir rock material loaded into a Viton tube until a length of 30 cm was achieved. Once compaction by radial confining pressure (30 bar(g)) was applied, methanol was injected until breakthrough, followed by brine, while collecting the effluent. Upon stable differential pressure and with at least 2 PV of brine injected, the effluent mixture was analyzed by means of nuclear magnetic resonance (NMR). Analyzing the mixture of brine and methanol helped to precisely identify the PV of the sandpack. Moreover, after obtaining this data, we could then calculate porosity and subsequently measure the permeability to brine of the sandpack.

Core Flooding Experiments—Sequence of Events: All slugs were injected at interstitial flow velocity of 1 ft/day. The following injection series format was used:

1. Synthetic formation brine (IWS-1010) injection up to ~1.6 pore volumes.
2. Chemical slug 1 injection up to 1 pore volume.
3. Chemical slug 2 injection up to 1.6 pore volumes.
4. Post-flush synthetic brine injection of approximately 4 pore volumes.

After performing the flooding experiments, core sample/sandpack was unloaded, and final saturations were confirmed via Dean–Stark measurement.

**Figure 5:**
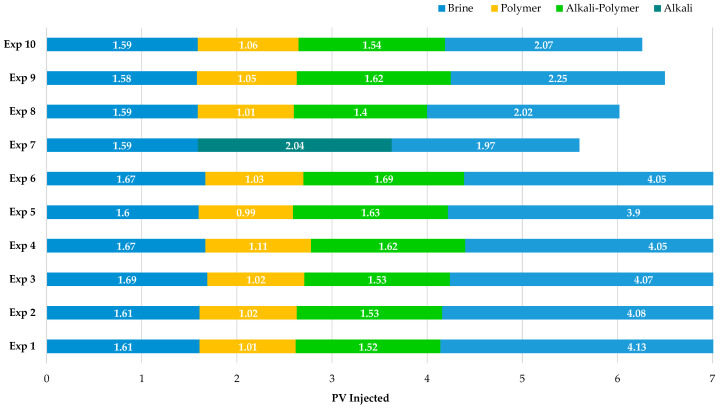
Overview of injected slugs in coreflooding experiments for outcrop (Experiment 1–Experiment 7) and sandpack (Experiment 8–Experiment 10) experiments. IWS-1010 brine was used for all indicated brine slugs.

Produced effluents were collected in 4.3 mL fractions and measurement of phase volumes was performed by visual metering of fluid levels. This was the case for oleic, emulsion, and aqueous phase volumes in the graduated fraction tubes. Elapsed time and differential pressure were recorded during the entirety of each experiment. While Table 5 and Table 6 summarize general experimental parameters and the used fluids, Figure 5 summarizes the injection sequences undertaken for each experiment.

## 4. Results & Discussion

**Figure 6:**
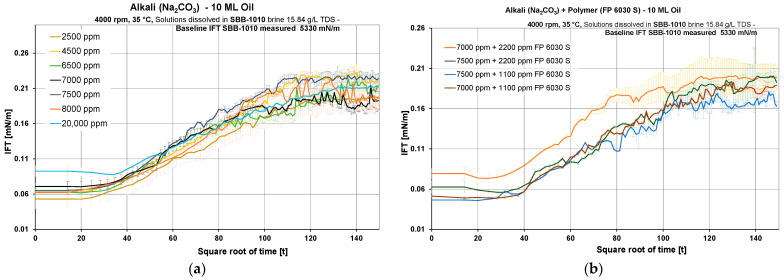
Interfacial tension data obtained for different concentrations of alkali: Na_2_CO_3_ and alkali–polymer: Na_2_CO_3_ + FP 6030 S reacting with oil 10-ML. Two areas are defined initial IFT from 0 to 20 sqt and equilibrium IFT 120–150 sqt. Baseline IFT for SBB-10. (**a**) alkali only. (**b**) alkali–polymer.

Interfacial Tension (IFT) and Phase Behavior Evaluations: Interfacial tension measurements indicated a 17-fold drop compared to the initial IFT obtained in an SBB-1010 and 10 ML oil system. This observation applies to all alkali and AP systems for the equilibrium IFT, as depicted in Figure 6a. The equilibrium interfacial tension (measured at the end of the experiment) ranged from 0.15 to 0.3 mN/m. The SBB-1010 brine has a lower interfacial tension (IFT) of 5.33 mN/m due to the presence of NaHCO_3_, in contrast to an average interfacial tension of around 27 mN/m in a brine/oil combination. We saw that IFT did not decrease any further when the alkali content increased. Introducing polymer into the solution, as depicted in Figure 6b, did not alter the observed interfacial tension behavior. Decreasing the polymer concentration in the AP solution from 2.2 g/L to 1.1 g/L resulted in a 15% reduction in interfacial tension (IFT), as shown by the blue line in Figure 6b. According to our understanding, polymers have both hydrophilic and hydrophobic groups, which govern the retention of produced surfactants through alkali. In our case, an increase in polymer concentration leads to a higher retention of produced surfactants, which, as a result, increases IFT. Overall, an average of 0.060 mN/m and 0.201 mN/m was observed in the spinning drop tensiometer for initial and equilibrium IFT, respectively, for all experiments.

Data obtained from phase behavior tests were used to determine IFT through the Chun–Huh equation (Liu et al. [31]) for alkali and AP systems, using a 10-mL oil sample (Table 7). We observed similar IFT values for the alkali and AP, with the latter being slightly lower. The IFT depicts a strong reduction, as observed in the spinning drop measurements.

After a detailed review of the phase behavior data in Table 7, it is evident that the top phase volumes exceeded 0.5 mL/mL. This suggests the potential creation of a water in oil (W/O) emulsion. In prior research [15], we have detected thermodynamically unstable emulsions with a comparable oil. Despite the instability of the emulsions, oil can be moved around the margins of viscous fingers due to the interaction of alkali with oil components and the creation of soaps at the oil/alkali solution interface. To make use of the described effect, fields can be selected with reactive oils suitable for alkali polymer flooding. For the case presented here, we also believe the high-water content in the oil makes it difficult to predict a proper phase behavior.

Micromodel Flooding Experiments: Micromodel flooding experiments were performed to quantify the recovery efficiency of combined polymer and AP prior to coreflooding. This provided a first screening of chemicals and fluid–fluid interactions. The results indicate that the brine composition has an impact on polymer viscosity, leading to a lower recovery factor (RF) when mixed in brine with divalent ions (IWS-1010). AP led to three times higher incremental RF compared with polymer alone, even when reducing polymer concentration in the AP slug. An overview of the results is shown in Table 8. Various of the observed mechanisms are presented in the following section.

**Figure 7:**
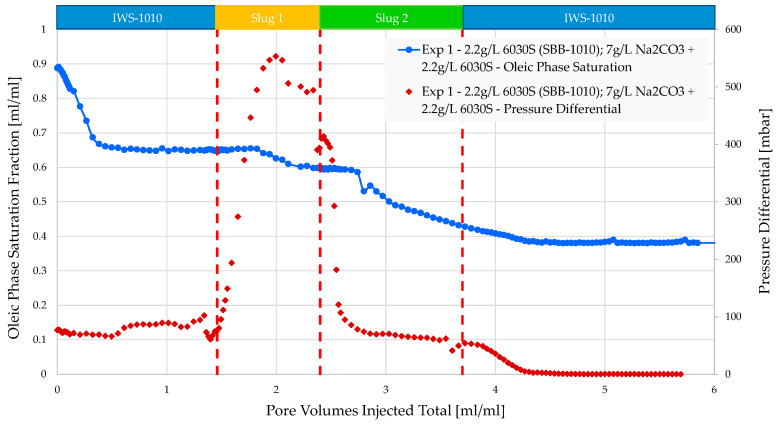
Oleic saturation, pressure differential vs. PV injected obtained for experiment 1 in micromodels. Slug 1 (orange area) refers to polymer alone and slug 2 (green area) to alkali–polymer.

**Figure 8:**
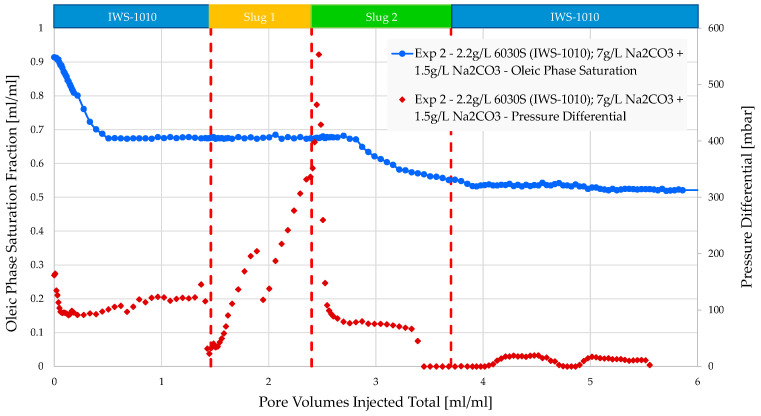
Oleic saturation, pressure differential vs. PV injected obtained for experiment 2 in micromodels. Slug 1 (orange area) refers to polymer alone and slug 2 (green area) to alkali–polymer.

The injection of brine in experiments 1 and 2 resulted in similar oil recovery factors of 27% and 26%, respectively. Figure 7 and Figure 8 show that the differential pressure behavior and magnitude for both remained consistent. Injecting polymer in experiment 1 resulted in a notably quicker rise in pressure difference and, overall, a more consistent plateau after breakthrough was achieved. In experiment 1, an extra relative permeability factor of 6% was noted. However, in experiment 2, there was minimal change, except for the repositioning of oil within the micromodel. This is the outcome of creating the polymer solution for experiment 1 in softened brine (SBB-1010) without divalent ions. The approach resulted in increased viscosities of the polymer solution, leading to a more favorable mobility ratio and an additional 6% recovery factor.

Alkali–polymer injection enhances injectivity and resulted in an extra RF of 21% in experiment 1 and 16% in experiment 2. The goal viscosity for both experiments (1–2) was selected to be equivalent to the viscosity of the polymer slug that was previously injected. In experiment 2, the polymer concentration in the AP slug was decreased to 1500 ppm, which is 700 ppm lower than in experiment 1. The post-flush with brine showed minimal impact on recovery; if there was any, this may relate to the remaining AP solution in the micromodel. However, injectivity could be further improved through the post-flush brine by washing out the AP solution.

**Figure 9:**
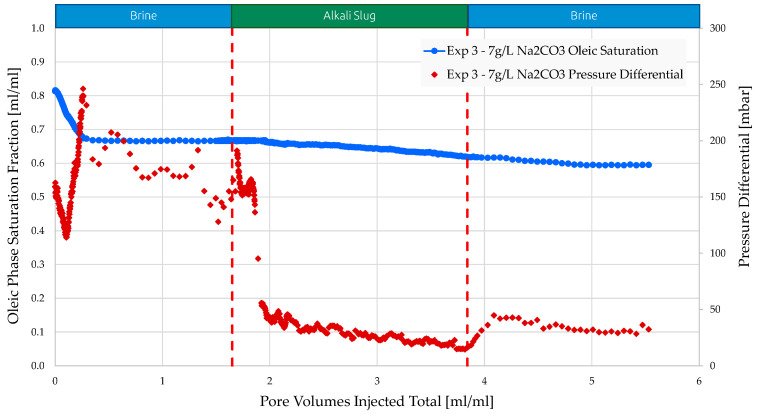
Oleic saturation, pressure differential vs. PV injected obtained for experiment 3 in micromodels.

In experiment 3 (Figure 9), injecting brine resulted in a reduced RF compared to experiments 1 and 2, due to the lower initial oil saturation. The brine injected had less oil to displace, leading to a reduced incremental recovery factor compared to experiment 1 and experiment 2. The differential pressure in experiment 3 was comparable to that of experiment 1 and experiment 2. Injecting alkali solution enhances injectivity by reducing differential pressure. The RF increased by just 6% over waterflooding. Oil was consistently and gradually produced during the entire alkali injection process, yet not effectively. Brine injection raised the differential pressure more significantly after injecting alkali compared to prior experiments. The picture analysis revealed that alkali injection is less efficient than AP. Alkali flows through the fingers and does not fully clean the pore space. When post-flush brine flows into the micromodel, it displaces the residual oil and the emulsion generated by alkali, causing a rise in pressure.

**Figure 10:**
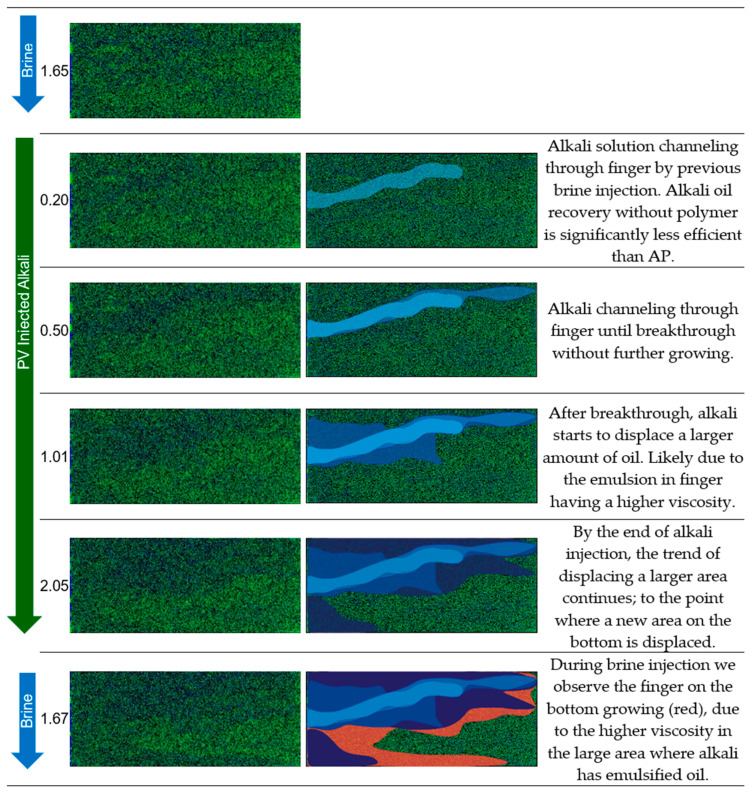
Displacement fronts during alkali and brine injection in experiment 3 (**left**: raw images after image segmentation; **right**: post-edit images highlighting displaced area). Green: oleic phase; blue: aqueous phase.

**Figure 11:**
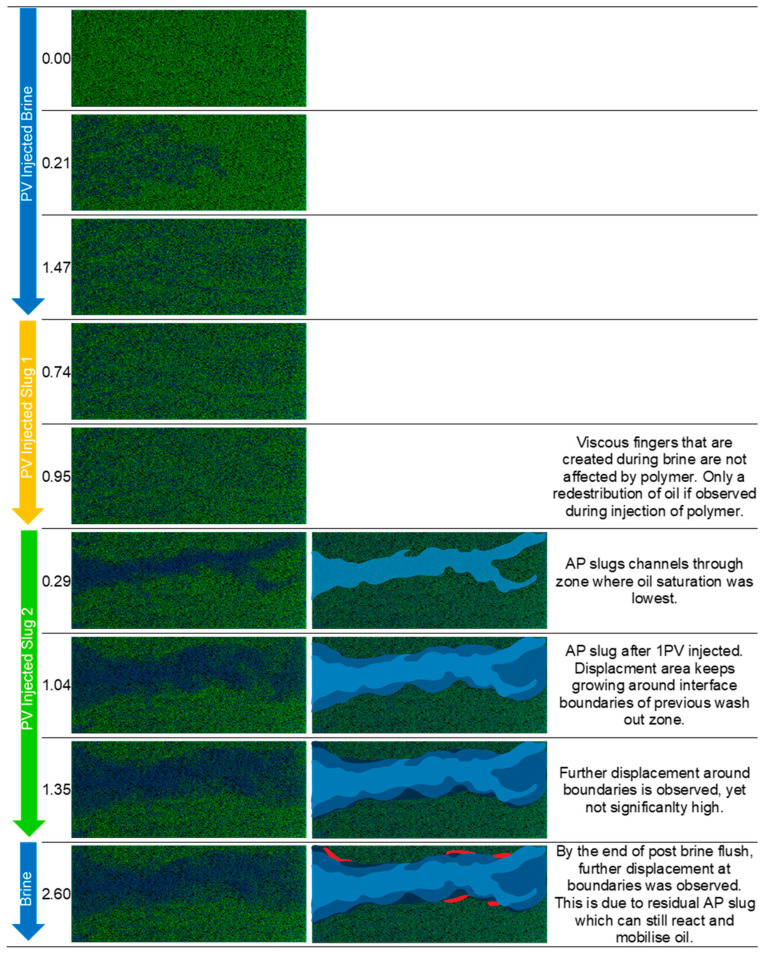
Displacement fronts during each slug injection in experiment 2 (**left**: raw images after segmentation; **right**: post-edit images highlighting displaced area). Green: oleic phase; blue: aqueous phase. Slug 1 refers to polymer and slug 2 refers to alkali–polymer.

**Figure 12:**
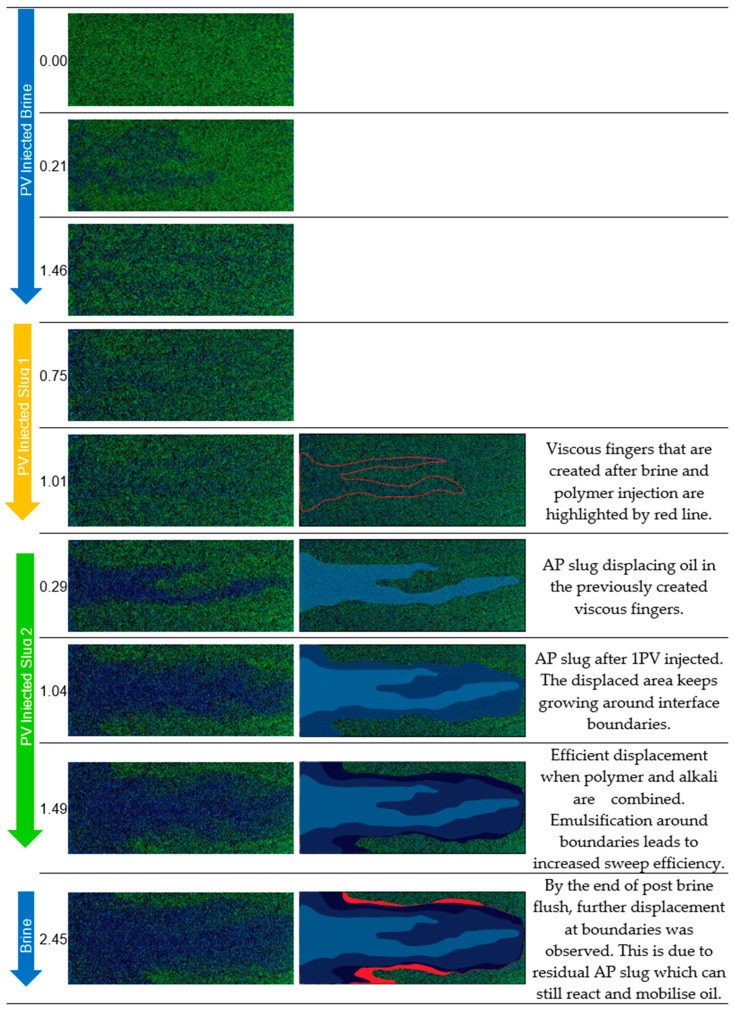
Displacement fronts during each slug injection in experiment 1 (**left**: raw images after analysis; **right**: post-edit images highlighting displaced area). Green: oleic phase; blue: aqueous phase. Slug 1 refers to polymer and slug 2 refers to alkali–polymer.

Displacement Mechanisms Observed During Micromodel Flooding: Micromodel applications enable visual access to the displacement process during injection. Hence, we could capture and analyze the behavior in each injected slug to better understand the mechanisms. We use experiment 3, experiment 2, and experiment 1 to illustrate displacement fronts and present a detailed description in Figure 10, Figure 11, and Figure 12, respectively.

**Figure 13:**
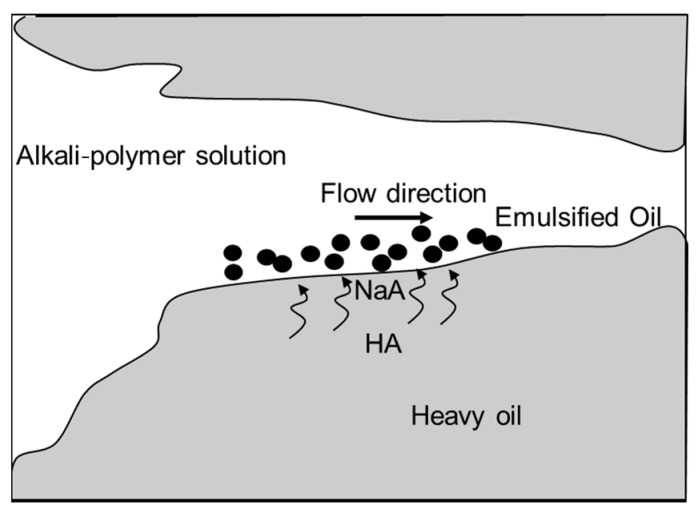
Schematic of the processes in alkali–polymer flooding of heavy oil. Alkali is saponifying components of the oil. The soaps are creating emulsions which are then moved through the viscous fingers.

In the case of heavy oil, water injection results in unstable displacement owing to the adverse water/oil mobility ratio (e.g., Hagoort [32], Kumar et al. [33], and de Loubens et al. [34]). Oil saturation at the core scale is influenced by viscous fingering, with bypassed oil not being displaced as highly mobile water dominates fluxes through the core, unlike heavy oil. Heavy oil frequently contains a significant number of components that can be saponified (HA) by the injection of alkali. A schematic for the displacement processes in heavy oil is shown in Figure 13. The components are diffusing towards the interface and converted with the alkali to soap (NaA), which is emulsifying the oil (e.g., Wu et al. [35] and Xiao et al. [23]). Polymers efficiently move emulsions through viscous fingers, generating new soap at the interface and moving emulsions in the flow direction. Fingers grow by continuously producing new soap at the interface and moving emulsions. In contrast to light or medium viscous oil, additional oil is not produced due to residual oil mobilized from the viscous finger boundary.

**Figure 14:**
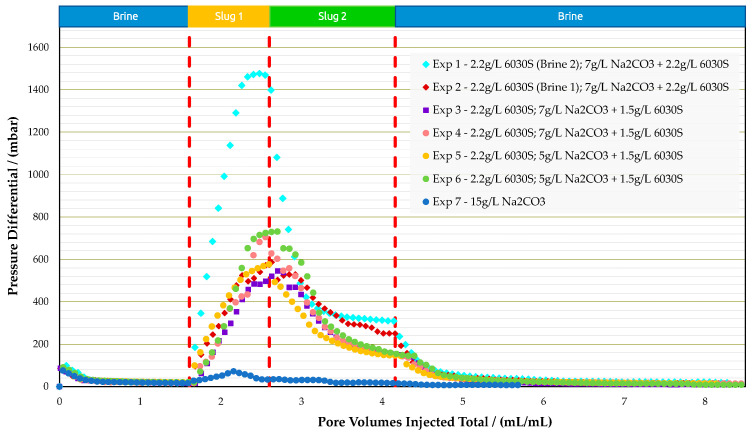
Summary of results oil saturation vs. pore volume obtained for experiments in Bentheimer outcrop core floods.

**Figure 15:**
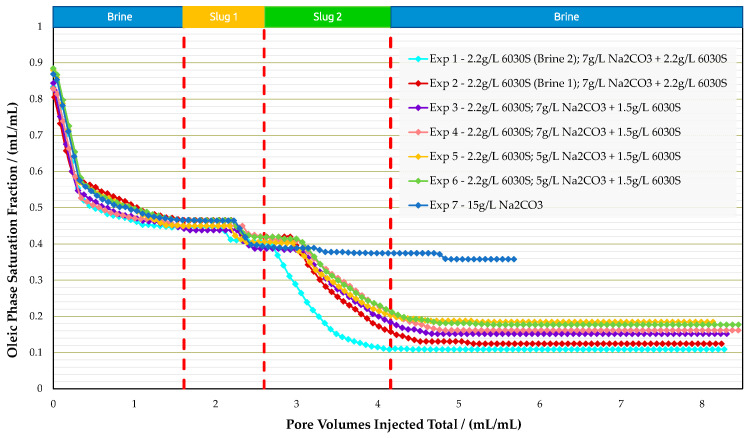
Summary of results pressure differential vs. pore volume obtained for experiments in Bentheimer outcrop core floods.

**Figure 16:**
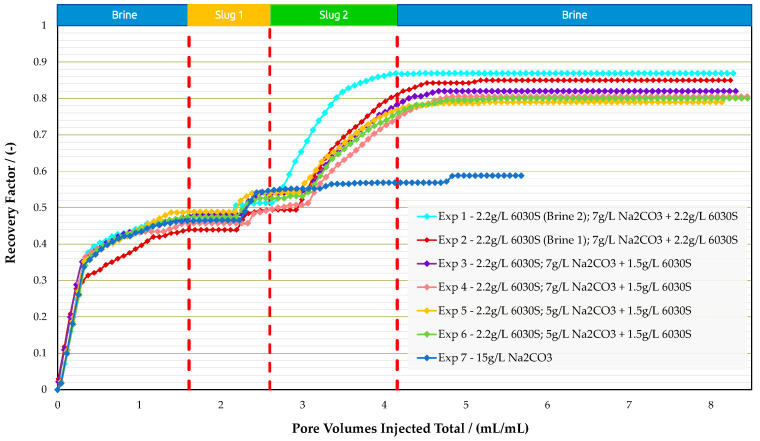
Summary of results recovery factor vs. pore volume obtained for experiments in Bentheimer outcrop core floods.

Coreflood Experiments in Bentheimer Outcrops: Micromodel experiments provided insights on how the AP slug behaves for the given oil–water system. Moreover, a reduction in polymer concentration by 700 ppm in the AP slug (from 2200 ppm in experiment 1 to 1500 ppm in experiment 2) worsened recovery by 5% compared with the higher polymer concentration. Hence, the coreflooding experiments were designed to investigate the economic feasibility of deploying chemical concentrations versus recovery efficiency. In each iteration, we improved the chemical formulation with respect to economics. A summary of the results is provided in Table 9 for the analogue sandstone experiments, including core properties. Accordingly, Figure 14, Figure 15, and Figure 16 summarize oil saturation, pressure differential, and recovery results for all analogue sandstone corefloods, respectively.

Experiments 1 and 2 were conducted to demonstrate the impact of divalent ions (Ca^2+^ and Mg^2+^) on polymer viscosity. Preparing the polymer slug in IWS-1010 reduced viscosity from 68 mPa·s to 32 mPa·s. Injectivity was enhanced from a differential pressure of 1500 mbar to approximately 580 mbar during the injection process. The same polymer at identical concentrations as those mentioned here is presently being injected in the Romanian field. The significant pressure difference observed in the core flood experiments suggests that induced fractures may be created under field settings, as documented in other studies (e.g., Shuaili et al. [36], Moe Soe Let et al. [37], and Zechner et al. [38]). Field applications of polymers have demonstrated that a significant portion of increased oil production is due to flow diversion rather than acceleration along flow paths. The Romanian field has seen alkali–polymer injection since 2023. Injectivity is as expected, and there has been a gradual increase in oil production.

The recovery of coreflooding experiments was in a similar range to micromodel experiments, where no additional recovery by the polymer was observed when the polymer was prepared in IWS-1010. AP injection significantly improved injectivity in both cases. From Table 9, one can derive that no additional oil recovery was obtained when the polymer viscosity doubled (chemical slug 1 at experiment 1 and experiment 2). We assume that the higher polymer viscosity is not high enough to substantially increase the capillary number. Thus, no additional oil recovery could be achieved for higher polymer viscosities. 

After determining that decreasing polymer viscosity had minimal effects on recovery during coreflooding, experiment 3 and experiment 4 were designed to align the viscosity of chemical slug 2 (alkali–polymer) with that of chemical slug 1 (polymer). Hence, we decreased the polymer concentration in the AP. These experiments were repeated to guarantee the reproducibility of results. The RF due to polymer remained the same as for experiment 1 and experiment 2. The AP slug with a decreased polymer concentration of 700 ppm resulted in slightly lower recovery values. The results indicate that cost optimization potential exists by decreasing polymer concentration during the AP slug, although this aspect was not included in the study. 

We conducted two more coreflooding tests, experiment 5 and experiment 6, to finalize the data set for this investigation. The alkali concentration in the AP slug was decreased from 7000 ppm to 5000 ppm in these studies. Our findings indicate that for an alkali concentration of 7 g/L, the incremental recovery for the AP slug after polymer injection averages 26.2%. Lowering the alkali concentration to 5 g/L reduces the incremental recovery after polymer injection to an average of 24.6%. The recovery mechanism caused by alkali in the AP slug can still produce a comparable level of extra recovery even with a 2 g/L reduction in alkali concentration. Lower alkali concentrations may be feasible; however, potential alkali losses from rock interaction must be considered during field implementation. 

One additional experiment was performed (experiment 7) where 15,000 ppm alkali (Na_2_CO_3_) without polymer was injected. We investigated and excluded any unwanted effects of alkali on the oil–rock system. Other than a slight pressure differential increase after approximately 0.6 PV of alkali injected, no abnormal behavior was observed. Displacement of oil was observed to be a continuous “wash-out”; with alkali being the emulsifying agent. If no polymer is present to improve mobility ratio, the alkali only emulsifies and shows poor incremental displacement. This is in alignment with what we observed in microfluidic experiments. The reason for using the high alkali concentration of 15 g/L was to investigate if any injectivity issues (rock–chemical interactions) occur during the injection of a 15 g/L alkali solution. In previous studies, we experienced an increase of differential pressure when injecting alkali only.

**Figure 17:**
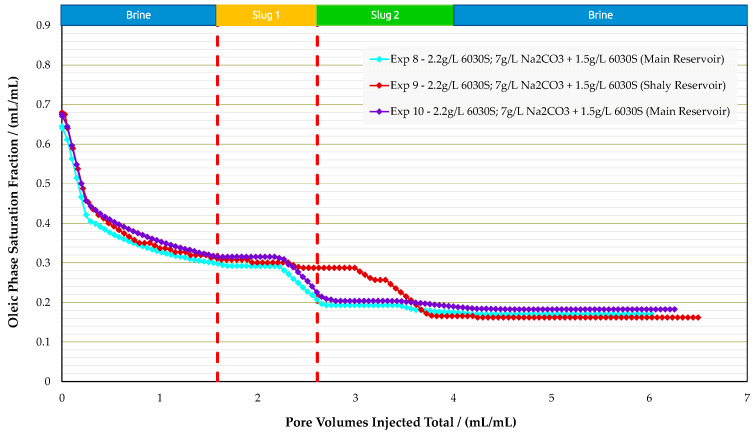
Summary of the results of oil saturation vs. pore volume obtained for experiments in sandpack floods.

**Figure 18:**
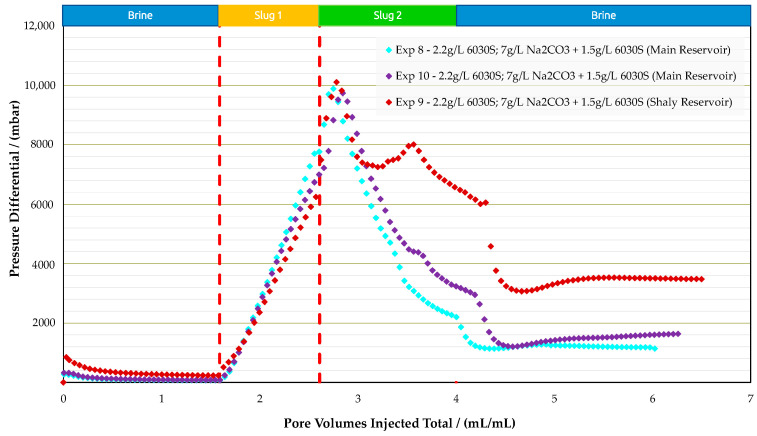
Summary of the results of pressure differential vs. pore volume obtained for experiments in sandpack floods.

**Figure 19:**
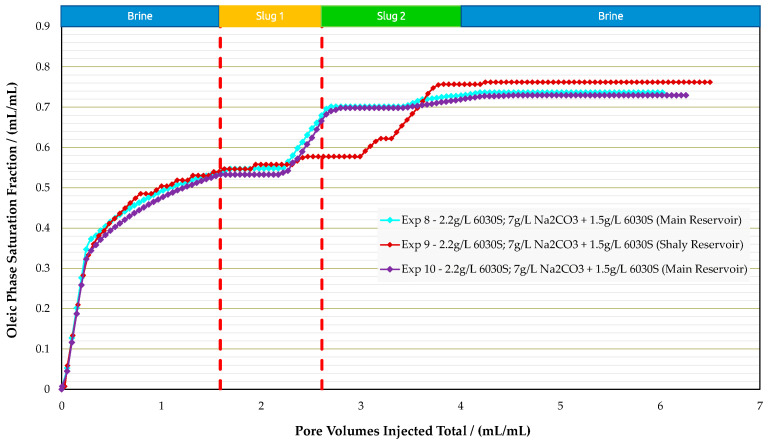
Summary of the results of recovery factor vs. pore volume obtained for sandpack floods.

Sandpack Flooding Experiments in Real Rock: Corefloods in analog rocks provided an estimate for the optimum concentrations; slug 1—polymer 2.2g/L 6030S—and slug 2—alkali-polymer 7.0g/L Na_2_CO_3_ + 1.5g/L 6030S. Two types of reservoir rock material were used to perform sandpack flooding experiments, namely the main reservoir and shaly reservoir. A summary of all three experiments is provided in Table 10. Note that in these experiments, permeability is an important parameter, since the main reservoir showed 1036 mD and 834 mD and the shaly reservoir 263 mD. Figure 17, Figure 18 and Figure 19 display oil saturation, pressure differential, and RF results for all sandpack floods.

Experiments performed in the main reservoir (experiment 8 and experiment 10) were designed with sandpacks matching a permeability of around 1 Darcy, as encountered in the field. The injection sequence was the same as for the analogue experiments. The pressure differential during water and polymer flooding increased significantly, which is in alignment with the lower permeability of the sandpacks compared to outcrop rocks. Once the AP slug was injected, we did not observe an immediate improvement in injectivity, as previously seen in analogue samples. On the contrary, the pressure differential increased by 5 bar, but after approximately 0.2 PV the injection pressure started to decrease as expected. 

Two experiments were performed with the main reservoir material, since in experiment 8, unexpected results were observed. We observed an incremental RF of 14% for the polymer slug, which was not seen in the analogue experiments. Performing an additional experiment at the same conditions led to similar results; experiment 10 (a repeat of experiment 8) showed the same recovery behavior. The polymer slug recovered more than the AP. This might be a result of lower S_or_ after polymer flooding compared to previous experiments. Thus, the AP slug had less oil volume to react with.

For the experiment with the shaly reservoir material (experiment 9), the performance was in alignment with the results for analogue core plugs. Polymer injection yielded low RF values and, once the AP slug was injected, the incremental recovery was 18.5%. The measured pressure differential values during the polymer slug were in the same range as for the main reservoir experiment, even though permeability was four times lower. This could be due to higher shear rates in the sandpack during injection. In this case, the sandpack contained fine grains, which were included during sandpack preparation to obtain lower permeability. The lower permeability of the shaly reservoir resulted in a higher in situ shear rate. Mathematical models in the literature report that the in situ shear rate is inversely proportional to permeability [39,40]. 

It is assumed that injected polymer slug was sensitive to in situ mechanical degradation while flowing through porous media. Hence, polymer flooding resulted in the same pressure drop as in experiments with higher permeabilities. However, higher pressure drops in the shaly formation, compared to the main reservoir sandpack during the initial water flooding, are in alignment with its low permeability. The experiments with the shaly reservoir rock reveal that clays could lead to additional effects related to their interaction with the injected solutions, such as the release of divalent ions from clay surfaces via cation exchange reactions (e.g., Strand et al. [41] and Bonto et al. [42]). Also, there might be negative effects of released calcium from clay surfaces on the phase behavior (Southwick et al. [43]), which need to be considered in a field implementation.

Resistance Factor (RF_i_): Based on the pressure response for each of the experiments, we have determined RF_i_. The values shown in Table 11 (outcrop floods) and Table 12 (sandpack floods) were determined by taking the final differential pressure (dP) point (mbar) of the respective slug and dividing it with the final dP point of the initial brine flood. RFf refers to the pressure drop at the end of the last brine injection divided by the pressure drop after the initial brine injection.

For experiments in Bentheimer outcrops, the obtained RF_i_ values averaged 30, with the exception of experiment 1 for polymer. The high observed RF_i_ is attributed to the high viscosity of the polymer slug (double) of experiment 1 compared with the other experiments (experiment 2–6). The data show that the polymer RF_i_s for experiment 8 and experiment 10 are much higher than the RF_i_s for experiment 2–6. The difference in rock composition is an important factor in the response of polymer RF_i_ for experiment 8 and experiment 10. RF_i_s for the AP slugs reached the highest value for experiment 1 in the case of the outcrop experiments. Much higher values of RF_i_ were seen for the case of the sandpack experiments than in the outcrop experiments. 

What stands out in Table 11 (outcrops floods) is the low RFfs for all experiments. This is attributable to the significant desaturation taking place in the cores. The low RFfs are a good indication that AP leads to limited permeability reduction; however, more detailed investigations are required. In contrast to those findings, a higher RFf was observed for the sandpack experiments. We attribute these higher values to the rock composition and the associated chemical adsorption, yet we consider them to be within the expected range.

Overall, Table 11 and Table 12 indicate that, among core floods, experiment 1 resulted in the highest RF_i_ and RFf due to the higher polymer slug viscosity. Further reduction of polymer slug viscosity owing to mixing with hard brine resulted in lower RF_i_s for both slugs and lower RFfs. This reduction in RF_i_ and RFf values due to lower polymer slug viscosity is further supported by the results of experiment 3. Furthermore, a reduction in the alkali concentration in experiment 6 compared to experiment 3 indicates that the RF_i_ and RFf for the AP slug remains the same. However, sole alkali injection without prior polymer injection showed the lowest RF_i_ due to poor mobility control and the absence of polymers. But, the RFf for alkali alone is similar to the values obtained for experiment 3 and experiment 6. This is an indication that the polymer adsorption, which might lead to a permeability reduction, is low in the case of AP injection for the outcrop cores. Combining incremental oil RF, RF_i_, RFf, and deployed chemical concentrations, experiment 5 and experiment 6 show the most promising results for application feasibility. In the sandpack experiments, the RF_i_s were very similar for the shaly and main reservoir case, although the permeabilities were very different. It seems as if a complex interplay of various effects, such as alkali–water–polymer–rock interaction and potential polymer shearing, is observed.

## 5. Economic Efficiency

Utility factors (UFs) refer to the chemical efficiency of the injected slugs (UF = kg chemicals injected/incremental oil production). For slugs consisting of one chemical, the UF can be used to compare economics. However, here, various chemicals were injected. To account for chemical agent’s costs, an equivalent utility factor (EqUF) can be calculated [44]:

$$\mathrm{EqUF}=\frac{\frac{m_{P}{\;*\;P}_{P}\;+\;m_{C}{\;*\;P}_{C}\;+\;m_{A}{\;*\;P}_{A}\;+\;\cdots \;\ldots \ldots }{P_{P}}}{N_{\mathrm{Pinc}}}\left[\frac{\mathrm{kg}}{\mathrm{bbl}}\right]$$

In the equation, “m” represents the mass in kilograms (kg) of each component injected, “P” stands for the price of a component in dollars per kilogram ($/kg), and “NPinc” denotes the incremental oil recovery in barrels (bbl). Subscripts “P”, “C”, and “A” are used for polymer, co-solvent, and alkaline, respectively. If additional components, such as a surfactant, are incorporated, the equation should be expanded to include “n” components. For this analysis, we assumed the cost of Na_2_CO_3_ to be $0.24/kg and the cost of polymer to be $2.5/kg. To simplify the comparison, our focus was on contrasting experiments conducted in outcrops. We assumed the injection of 1 pore volume (PV) of alkaline polymer (AP), with the resulting incremental oil recovery from the AP slug. In the laboratory, we injected 1.5 PV to ensure complete displacement by the AP. However, it is worth noting that injecting more than 1 PV should not be necessary, even when considering dispersion. Therefore, we utilized 1 PV for the one-dimensional EqUF calculation.

**Figure 20:**
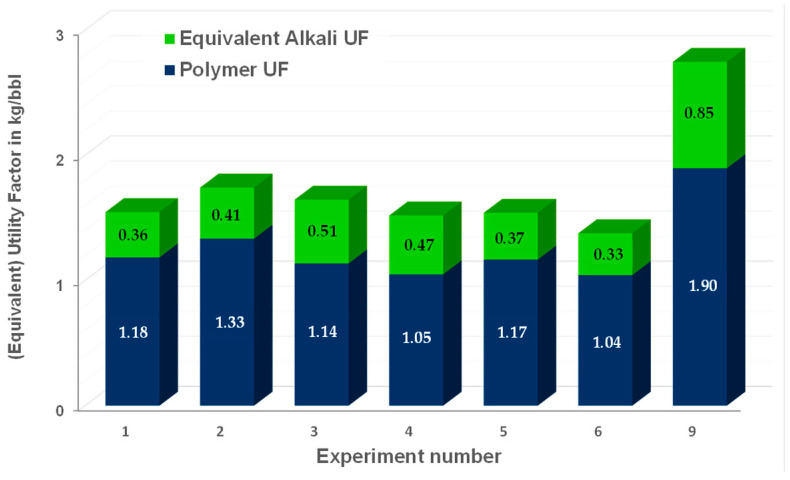
Equivalent utility factor (EqUF) for selected experiments. The EqUF shown here reflects the incremental oil produced by the alkali–polymer slug and the mass of chemicals injected during this slug, assuming 1 PV injection.

The EqUFs for injection of the aged and non-aged polymers with alkali for experiment 1–6 and experiment 9 are shown in Figure 20. It is shown that higher EqUFs are observed for experiment 9 conducted in real rock on the shaly area. The observed EqUFs for experiments in the Bentheimer outcrops are very similar to each other. Reducing the polymer concentration in experiment 3 led to a very small decrease in EqUF, by 5%. We have not tested injecting AP immediately without a prior polymer slug.

We observe a strong indication that initiating the injection of AP promptly would be advantageous for the project. Although the testing area in the chosen Romanian field is currently undergoing polymer flood operations for operational reasons, the implementation will occur in a section of the field that has not yet been subjected to polymer injection.

## 6. Summary and Conclusions

We have taken into account the physicochemical mechanisms and the displacement efficiency through alkali and alkali–polymer flooding techniques in heavy oil contexts. This exploration included assessments under dynamic flooding conditions via core floods and micromodels, backed by insights from interfacial tension (IFT) and phase behaviors. Our investigation covered the impacts at the oil–water interfaces, the efficiency of displacement, and, notably, the function of polymers in these contexts. The key findings include:

- Experiments indicated that alkali treatments initially reduce IFT and maintain favorable equilibrium IFT with reactive viscous oils, attributed to the gradual formation of soap at the oil–alkali interface, due to diffusion.
- Alkali injection into reactive viscous oils showed limited effectiveness in lowering residual oil saturation. Observations in micromodels revealed minor oil mobilization at the fringes of viscous fingers. However, alkali–polymer (AP) flooding significantly enhanced oil recovery by effectively transporting mobilized oil within the viscous fluid, thereby exposing more oil to the alkali treatments.
- We introduced novel insights into the pore-scale mechanisms occurring during polymer and alkali–polymer deployment.
- Core flood experiments confirmed the micromodel observations, showing that reducing polymer concentrations to match the viscosity of solutions with divalent cations achieved similar displacement efficiencies.
- Favorable recoveries were also documented using reservoir rock materials and sandpacks, suggesting the potential efficiency of these processes.
- Significant pressure drops were observed during polymer injections in core floods, which, in field conditions, could induce fractures and alter flow paths. Conversely, AP injections exhibited lower pressure drops, potentially enhancing field injectivity.
- Sandpack flooding tests indicated that clay interactions might introduce additional factors, such as cation exchange and the effects of released ions on phase behavior, which are crucial for field application considerations.
- The economic viability of AP flooding projects could be enhanced by optimizing polymer concentrations in the AP slug with softened water, achieving an optimal balance between reduced polymer concentrations and the benefits of increased viscosities in water with fewer divalent cations, alongside improved recovery factors.
- Future directions necessitate further research into managing produced emulsions and more detailed investigations on the effects of alkali–rock interactions.

## Figures and Tables

**Table 1:** Crude oil composition (reservoir ML, Well 10-ML). Saponifiable acids using titration.

Property	Value
TAN [mg KOH/g]	5.67
Saturates [%]	33
Aromatics [%]	33
Resins [%]	18
Asphaltene [%]	16
Saponifiable Acids [µmol/g]	116
ρ @ 22 °C [g/cm^3^]	0.953

**Table 2:** Composition of brines used in this study.

Salt	IWS-1010 (g/L)	SBB-1010 (g/L)
NaCl	13.00	14.56
KCl	0.04	-
MgCl_2_·6H_2_O	1.60	-
CaCl_2_·2H_2_O	1.20	-
NaHCO_3_	-	0.46
TDS	15.84	15.02

**Table 3:** Mineral composition of core material used in experiments. Units in weight percent.

Core	Quartz	Kaolinite	K-F_sp_ *	Calcite	Microcline	Dolomite	Clay Tot.
Bentheimer Outcrop	97.3	1.1	-	-	1.2	0.4	-
Main Reservoir	51.26	20.1	17.86	1.54	-	1.48	7.76
Shaly Reservoir	41.73	18.7	19.41	10.04	-	0.32	9.8

* K-F_sp_ = Potassium feldspar.

**Table 4:** Summary of micromodel experiment parameters performed in this work. Pore pressure was 1 bar, injection rate 1 ft/day, and temperature 35 °C in all cases.

Parameter	Units	Experiment 1	Experiment 2	Experiment 3
Chemical Slug 1	-	2.2 g/L 6030S *	2.2 g/L 6030S **	7g/L Na_2_CO_3_ *
Viscosity Slug 1	mPa·s	68	32	0.81
Chemical Slug 2	-	7g/L Na_2_CO_3_ + 2.2 g/L 6030S *	7g/L Na_2_CO_3_ + 1.5 g/L 6030S *	-
Viscosity Slug 2	mPa·s	63	32	-

* Diluted in synthetic brine SBB-1010: 14.56 g/L NaCl and 0.46 g/L NaHCO_3_. ** Diluted in synthetic brine IWS-1010: 13 g/L NaCl, 0.04 g/L KCl, 1.2 g/L CaCl_2_·2H_2_O, and 1.6 g/L MgCl_2_·6H_2_O.

**Table 5:** Chemical concentrations of individual slugs and their respective viscosities used for Bentheimer outcrop flooding experiments. Core orientation was vertical, pore pressure 5 bar, core injection velocity 1 ft/day, radial pressure of 30 bar, and temperature 35 °C in all cases.

Parameter	Unit	Experiment 1	Experiment 2	Experiment 3 & 4	Experiment 5 & 6	Experiment 7
Chem. Slug 1	-	2.2 g/L 6030S *	2.2 g/L 6030S **	2.2 g/L 6030S **	2.2 g/L 6030S **	-
η Slug 1 (7.94 s^−1^)	mPa·s	68	32	32	32	-
Chem. Slug 2	-	7 g/L Na_2_CO_3_ + 2.2 g/L 6030S *		7 g/L Na_2_CO_3_ + 1.5 g/L 6030S *	5 g/L Na_2_CO_3_ + 1.5 g/L 6030S *	15 g/L Na_2_CO_3_ *
η Slug 2 (7.94 s^−1^)	mPa·s	63	63	32	31	0.81

* Diluted in synthetic brine SBB-1010: 14.56 g/L NaCl and 0.46 g/L NaHCO_3_. ** Diluted in synthetic brine IWS-1010: 13 g/L NaCl, 0.04 g/L KCl, 1.2 g/L CaCl_2_·2H_2_O, and 1.6 g/L MgCl_2_·6H_2_O.

**Table 6:** Chemical concentrations of individual slugs and respective viscosities used for sandpack flooding experiments. Core orientation was vertical, pore pressure 5 bar, core injection velocity 1 ft/day, radial pressure of 30 bar, and temperature 35 °C in all cases.

Parameter	Unit	Experiment 8	Experiment 9	Experiment 10
Chemical Slug 1	-	2.2 g/L 6030S **		
η Slug 1 (7.94 s^−1^)	mPa·s	38	41	39
Chemical Slug 2	-	7 g/L Na_2_CO_3_ + 1.5 g/L 6030S *		
η Slug 2 (7.94 s^−1^)	mPa·s	36	34	34

* Diluted in synthetic brine SBB-1010: 14.56 g/L NaCl and 0.46 g/L NaHCO_3_. ** Diluted in synthetic brine IWS-1010: 13 g/L NaCl, 0.04 g/L KCl, 1.2 g/L CaCl_2_·2H_2_O, and 1.6 g/L MgCl_2_·6H_2_O.

**Table 7:** Overview of phase behavior tests and calculated IFT using the Chun–Huh equation (Liu et al. 2008 [31]) for alkali and alkali–polymer, performed with 10-ML oil sample. IFT = V middle phase/V lower phase × c, c = 0.3 nM/m (Liu et al. 2008 [31]). Lower phase = water, middle phase = emulsion, upper phase = oil. Data is shown for A = 7.0 g/L Na_2_CO_3_ and AP = 7.0 g/L Na_2_CO_3_ + 2.2 g/L FP 6030 S.

	Time [Days]	Lower Phase (LP) Vol. [ml/mL]	Middle Phase (MP) Vol. [ml/mL]	Upper Phase (UP) Vol. [ml/mL]	$\mathrm{Ratio}\;\frac{V_{Middle\;Phase}}{V_{Lower\;Phase}}$ [-]	IFT MP/LP [mN/m]
A	6	0.10	0.01	0.89	0.100	0.0300
	21	0.12	0.02	0.86	0.167	0.0500
	45	0.14	0.03	0.83	0.214	0.0643
	66	0.16	0.03	0.81	0.188	0.0563
	83	0.17	0.03	0.80	0.174	0.0522
	104	0.19	0.03	0.78	0.154	0.0462
	127	0.23	0.04	0.73	0.174	0.0522
	140	0.24	0.08	0.68	0.313	0.0938
AP	6	0.173	0.01	0.817	0.058	0.017
	21	0.190	0.02	0.790	0.105	0.032
	45	0.220	0.03	0.750	0.136	0.041
	66	0.237	0.03	0.733	0.127	0.038
	83	0.267	0.03	0.703	0.113	0.034
	104	0.280	0.03	0.690	0.107	0.032
	127	0.318	0.04	0.642	0.126	0.038
	140	0.347	0.075	0.578	0.216	0.065

**Table 8:** Micromodel flooding parameters and results. Pore pressure was 1 bar injection rate 1 ft/day and temperature 35 °C.

Parameter	Units	Experiment 1	Experiment 2	Experiment 3
Polymer (P)	-	2.2 g/L 6030S *	2.2 g/L 6030S **	-
η Polymer (7.94 s^−1^)	mPa·s	68	32	-
Alkali-polymer (AP)	-	7g/L Na_2_CO_3_ + 2.2 g/L 6030S *	7g/L Na_2_CO_3_ + 1.5 g/L 6030S *	7g/L Na_2_CO_3_ *
η AP (7.94 s^−1^)	mPa·s	63	32	0.81
So initial	%	89	91	81
So final	%	38	52	60
R (Brine)	%	27	26	18
Add. R (P)	%	6	0	-
Add. R (AP)	%	21	16	-
Add. R (A)	%	-	-	6
Add. R (Brine)	%	3	1	3

* Diluted in synthetic brine SBB-1010: 14.56 g/L NaCl and 0.46 g/L NaHCO_3._ ** Diluted in synthetic brine IWS-1010: 13 g/L NaCl, 0.04 g/L KCl, 1.2 g/L CaCl_2_·2H_2_O and 1.6 g/L MgCl_2_·6H_2_O.

**Table 9:** Results and parameters for Bentheimer outcrop flood experiments. Core orientation was vertical, pore pressure 5 bar, injection rate 1 ft/day, radial pressure 30 bar, and temperature 35 °C in all cases. Viscosities are taken at 7.94 s*^−^*^1.^

Parameter	Unit	Experiment 1	Experiment 2	Experiment 3	Experiment 4	Experiment 5	Experiment 6	Experiment 7
Slug 1	g/L	2.2 P *	2.2 P **	2.2 P **	2.2 P **	2.2 P **	2.2 P **	-
η Slug 1	mPa·s	68	32	32	32	32	32	-
Slug 2 *	g/L	7.0 A + 2.2 P		7.0 A + 1.5 P		5.0 A + 1.5 P		15.0 A
η Slug 2	mPa·s	63	63	32	32	31	31	0.81
Length	cm	29.80	29.80	29.80	30.15	30.20	30.20	30.30
Diameter	cm	2.96	2.96	2.96	2.96	2.96	2.96	2.96
Bulk Volume	cm^3^	203.83	203.63	203.82	207.47	207.82	207.62	206.56
PV	cm^3^	47.00	46.73	45.42	47.71	49.43	49.46	49.68
Porosity	%	23.30	23.85	23.80	24.30	23.90	23.90	24.00
Perm. (kw)	mD	2258	2223	2283	2530	2150	2253	2104
Oil Sat. Init.	%	83	83	84	83	87	89	87
R brine	%	46.50	43.90	48.10	45.70	48.90	47.40	46.4
Additional Recovery Polymer	%	4.80	5.50	6.00	4.50	5.10	5.20	-
Additional Recovery AP	%	35.60	31.60	25.00	27.40	23.50	25.70	10.30
Add. Rec. Brine	%	0.00	4.00	2.90	3.00	2.00	0.00	2.10

* Diluted in synthetic brine SBB-1010: 14.56 g/L NaCl and 0.46 g/L NaHCO_3._ ** Diluted in synthetic brine IWS-1010: 13 g/L NaCl, 0.04 g/L KCl, 1.2 g/L CaCl_2_·2H_2_O and 1.6 g/L MgCl_2_·6H_2_O. P = 6030S. A = Na_2_CO_3._

**Table 10:** Results and parameters for sandpack flooding experiments. Core orientation was vertical, pore pressure 5 bar, injection rate 1 ft/day, radial pressure 30 bar, and temperature 35 °C in all cases.

Parameter	Unit	Experiment 8	Experiment 9	Experiment 10
Chemical Slug 1	-	2.2 g/L 6030S **		
Ƞ Slug 1 (7.94 s^−1^)	mPa·s	38	41	39
Chemical Slug 2	-	7g/L Na_2_CO_3_ + 1.5 g/L 6030S *		
Ƞ Slug 2 (7.94 s^−1^)	mPa·s	36	34	34
Length	cm	29.45	29.45	29.55
Diameter	cm	2.99	2.99	2.99
Bulk Volume	cm^3^	206.68	206.68	207.38
Pore Volume	cm^3^	72.00	66.00	70.00
Porosity	%	34.80	31.90	33.80
Brine perm. (kw)	mD	1036	263	834
Init. Oil Saturation	%	65	68	68
Recovery brine	%	53.80	53.90	53.30
Additional Recovery Polymer	%	14.10	3.80	14.90
Additional Recovery AP	%	5.00	18.50	3.60
Additional Recovery Brine	%	0.60	0.00	0.20

* Diluted in synthetic brine SBB-1010: 14.56 g/L NaCl and 0.46 g/L NaHCO_3._ ** Diluted in synthetic brine IWS-1010: 13 g/L NaCl, 0.04 g/L KCl, 1.2 g/L CaCl_2_·2H_2_O, and 1.6 g/L MgCl_2_·6H_2_O.

**Table 11:** Summary of calculated resistance factor (RF_i_) and resistance factor after the final brine injection (RFf) from outcrop flood experiments in this work. Table shows differential pressure taken for the RFi and RRf calculation.

	Slug	Experiment 1	Experiment 2	Experiment 3	Experiment 4	Experiment 5	Experiment 6	Experiment 7
RF_i_	P	82.23 (1398/17)	32.55 (586/18)	34.13 (546/16)	32.18 (547/17)	28.85 (577/20)	36.55 (731/20)	-
	AP	18.17 (309/17)	13.88 (250/18)	9.36 (150/16)	6.18 (105/17)	7.25 (145/20)	7.25 (145/20)	-
	A	-	-	-	-	-	-	1.13 (18/16)
RFf	-	2.00 (34/17)	0.83 (15/18)	0.57 (9/16)	0.88 (15/17)	0.75 (15/20)	0.45 (9/20)	0.44 (7/16)

**Table 12:** Summary of calculated resistance factor (RF_i_) and resistance factor after the final brine injection (RFf) from sandpack flood experiments performed in this work. Table shows differential pressure taken for the RF_i_ and RRf calculation.

	Slug	Experiment 8	Experiment 9	Experiment 10
RF_i_	P	110.94 (7766/70)	31.87 (7491/235)	89.09 (7216/81)
	AP	31.38 (2197/70)	25.55 (6005/235)	36.37 (2949/81)
RFf	-	16.2 (1139/70)	14.8 (3479/235)	20.19 (1635/81)

## Data Availability

Data are contained within the article.

## Abbreviations

AP	Alkali Polymer
ASP	Alkali Surfactant Polymer
EOR	Enhanced Oil Recovery
IFT	Interfacial Tension
HA	Saponifiable components in the oil
NaA	Soap
NMR	Nuclear magnetic resonance
PV	Pore Volume
RF	Recovery Factor
RF_i_	Resistance Factor
RF_f_	Residual Factor after the last brine flood
TAN	Total Asset Number
TVD	True Vertical Depth
